# Unmasking the risk: clinical trials *versus* real-world evidence on dydrogesterone and birth defects

**DOI:** 10.1093/hropen/hoae073

**Published:** 2024-12-24

**Authors:** Fabio Parazzini, Anna Cantarutti, Giovanna Esposito

**Affiliations:** Department of Clinical Sciences and Community Health, Dipartimento di Eccellenza 2023-2027, University of Milan, Milan, Italy; Unit of Biostatistics, Epidemiology and Public Health, Department of Statistics and Quantitative Methods, University of Milano-Bicocca, Milan, Italy; National Centre for Healthcare Research and Pharmacoepidemiology, Milan, Italy; Department of Clinical Sciences and Community Health, Dipartimento di Eccellenza 2023-2027, University of Milan, Milan, Italy

The use of progesterone for luteal phase support and the treatment of threatened miscarriage in early- to mid-pregnancy has been an established practice for decades (Haas *et al.*, 2018). It is commonly administered via oral, intramuscular, or intravaginal routes. In the context of medically assisted reproduction procedures, the use of micronized vaginal progesterone has largely replaced the use of intramuscular progesterone to avoid the unpleasant effects of intramuscular administration. On the other hand, vaginal administration also causes burning, irritation, and secretions. Thus, oral administration may represent the route of choice. However, the administration of oral micronized progesterone for luteal phase support in IVF is limited by its reduced bioavailability and substantial first-pass metabolism. In contrast, dydrogesterone, a stereoisomer of progesterone, shows high efficacy at low doses, due to its oral bioavailability and receptor specificity, making it an attractive oral alternative to micronized vaginal progesterone. With potent progestogenic activity and high selectivity for progesterone receptors, it shows negligible or no agonistic activity at androgen, glucocorticoid, and mineralocorticoid receptors (Griesinger *et al.*, 2019).

Over the past 10 years, several randomized clinical trials (Chakravarty *et al.*, 2005; Ganesh *et al.*, 2011; Tomic *et al.*, 2015; Saharkhiz *et al.*, 2016; Tournaye *et al.*, 2017; Griesinger *et al.*, 2018) have evaluated the efficacy and safety of oral dydrogesterone and micronized vaginal progesterone in supporting the luteal phase during IVF procedures. In 2020, Griesinger *et al.* conducted a systematic review and meta-analysis of individual participant data from ∼2000 subjects to compare the efficacy of oral dydrogesterone and micronized vaginal progesterone (Griesinger *et al.*, 2020). The analysis demonstrated a significant difference between the two treatment modalities. Specifically, the probability of pregnancy was higher with oral dydrogesterone (odds ratio (OR) = 1.16, 95% CI: 1.01–1.34), as was the probability of live birth (OR = 1.19, 95% CI: 1.03–1.38) (Griesinger *et al.*, 2020). This evidence has led to a growing trend toward the use of dydrogesterone in medically assisted reproduction to support the luteal phase.

However, a background noise has always accompanied the use of progesterone, particularly dydrogesterone, during pregnancy. Since the 1960s, some reports have suggested a possible teratogenic effect of exposure to progesterone during pregnancy, especially on the risk of hypospadias and the development of congenital heart disorders (Heinonen *et al.*, 1977). This topic was of great interest in the 1980s and disappeared from the literature because the product was no longer used during early pregnancy. The few papers published during the last 30 years have shown inconsistent findings. Recently, a systematic review, including six randomized controlled trials, one case-control study, one prospective, and one retrospective cohort study, showed no significant differences in the risk of congenital anomalies after exposures to dydrogesterone *versus* progesterone or micronized vaginal progesterone. However, the certainty of the evidence, according to the Grading of Recommendation Assessment, Development and Evaluation (GRADE) approach, was judged as low (Katalinic *et al.*, 2024).

In a study published in this issue of *Human Reproduction Open*, Henry *et al.* (2024) have analyzed the risk of congenital heart malformation following intake of dydrogesterone in medically assisted reproduction procedures using data that was collected as part of the post-marketing pharmacovigilance from the World Health Organization (WHO) global database, VigiBase. The authors analyzed the frequency of reports of malformations in women who had used dydrogesterone and compared this frequency with that reported in the database for any other drug, other drugs specifically used for ART, and progesterone. They reported: ‘*A significantly higher disproportionate reporting of birth defects was found with dydrogesterone when compared to any other drug (ROR [reporting odds ratio] 5.4, 95%CI [3.9–7.5]), to any other ART drug (ROR 6.0, 95%CI [4.2–8.5]) and to progesterone (ROR 5.4, 95%CI [3.7–7.9]). Major birth defects reported with dydrogesterone were mainly genital defects such as hypospadias and congenital heart defects*’.

Therefore, Henry *et al.* (2024) have successfully reopened the debate by specifically focusing attention on the current indications for the use of dydrogesterone in medically assisted procreation procedures. The authors correctly underline that these data must be considered with caution.

## What is pharmacovigilance?

Pharmacovigilance is a scientific discipline that encompasses detecting, assessing, understanding, and preventing adverse drug reactions and other drug-related safety issues. It involves continuously monitoring the safety profile of medicinal products to ensure that their benefits outweigh their risks.

The thalidomide tragedy of the early 1960s, which led to severe birth defects in ∼10 000 children exposed to thalidomide during pregnancy, highlighted the necessity for rigorous post-marketing drug safety surveillance. In response, the WHO established VigiBase, which collects individual case safety reports (ICSRs) from more than 150 national pharmacovigilance centers worldwide. Each ICSR includes information about the adverse reaction(s), patient demographics, drug data, and outcome. ICSRs can be filed by healthcare workers, patients, and pharmaceutical firms.

Pharmacovigilance is undoubtedly a fundamental tool in the post-marketing evaluation of the safety of a drug. Pharmacovigilance data is a type of real-world data that is a valuable complement to randomized controlled trials. While they may not provide the same level of detail about a drug’s effectiveness, they offer important advantages, such as longer observation periods (some adverse events—for example, some congenital malformations—can be diagnosed late, i.e. after the end of the observation period foreseen in controlled clinical trials) and insights from larger and more representative populations, including people who may not be eligible for clinical trials because of their age, pre-existing conditions or concomitant medications. In the case of rare events, no drug is substantially marketed with adequate support due to the rarity of the event in the subjects studied in the registration studies.

This broader scope makes pharmacovigilance data particularly useful for understanding how medicines work in everyday clinical practice. However, many cautions must be considered when interpreting these results.

The first traditionally well-recognized limitation of the spontaneous reporting system used for pharmacovigilance is underreporting. This limit leads to a potential underestimation of the true risk associated with a drug. If underreporting disproportionately affects mild or moderate adverse events, reports may be biased toward more serious or unusual cases, inevitably leading to overestimation of the frequency or significance of specific adverse events if the denominator is the total number of adverse reports. Underreporting can occur differently depending on the drug and the existing knowledge about its safety profile. In fact, it is conceivable that an adverse event already documented is more likely to be reported than the same event that occurred after exposure to a drug with no reported association in the literature. This potential reporting bias may, in part, be suggested by the observation that the frequency of non-malformative adverse events appears to be lower in the Henry *et al.* (2024) study after exposure to dydrogesterone than other drugs, including progesterone.

Other limitations of the pharmacovigilance system based on spontaneous reports are the confounding factors, variation in reporting practices and lack of clinical data, and the total number of exposed subjects. Thus, in the study by Henry *et al.* (2024), the denominator considered was not the number of women exposed to dydrogesterone, but the number of reports of adverse events for its use. If exposure to progesterone alone were to cause more non-malformative adverse events, this would reduce the rate of malformative events compared to that observed with dydrogesterone. Additionally, different prevalences of the pathology reported in spontaneous reports may be due to some populations having a tendency to overestimate or underestimate an association, especially if the comparator drug is used in a dissimilar way in diverse populations. For example, the frequency of hypospadias is higher in the USA (Yu *et al.*, 2019), a country where the number of spontaneous reports related to dydrogesterone use appears to be very limited. Finally, pharmacovigilance systems generally focus on the relationship with a single drug. However, exposure in general in clinical practice is not limited to a single drug; for example, women exposed to dydrogesterone/progesterone would also be taking hCG at the same time (Manickavasagam *et al.*, 2024).

The results of retrospective (Zaqout *et al.*, 2015) or pharmacovigilance studies, however, are in contrast with the evidence of controlled clinical studies. The previously quoted systematic review and meta-analysis (Griesinger *et al.*, 2020) considered the risk of malformations, finding a similar incidence of congenital, familial, and genetic disorders for oral dydrogesterone *versus* micronized vaginal progesterone use. Other reviews and meta-analyses focusing on the safety data for dydrogesterone *versus* progesterone use found that the pooled risk ratios for fetal abnormalities were close to 1, supporting no causal association between fetal abnormalities and first-trimester dydrogesterone use (Katalinic *et al.*, 2022, 2024).

Both clinical studies and pharmacovigilance data do not analyze the aforementioned role of potential drug interactions. Similarly, the role of different doses and intake schedules of dydrogesterone are not easily assessable in the context of pharmacovigilance studies. Dydrogesterone is used in different doses (from 20 to 40 mg per day) and duration of use (from the day of the oocyte retrieval or embryo transfer until the 10th or 12th week of gestation) (Griesinger *et al.*, 2020; Manickavasagam *et al.*, 2024). The role of different usage patterns on the risk of malformations may be important and needs careful evaluation compared with the potential benefits of dydrogesterone.

In general, the potential biological mechanism of a relationship between dydrogesterone intake during pregnancy and the risk of malformations is not known. It is possible to hypothesize that, given the activity of dydrogesterone on the pathways of androgenesis, its intake could lead to a decrease in the production or action of androgens, triggering isolated cases of hypospadias. It is more complex to hypothesize the mechanism associated with the risk of cardiac malformation pathology.

In medicine, we cannot always use the ‘Once an accident, twice a coincidence, three times a pattern’ criterion. However, the study by Henry *et al.* (2024) brings the potential issues related to the use of dydrogesterone during pregnancy to clinicians’ attention once again. Despite the abovementioned limitations, the pharmacovigilance analysis suggests a greater risk for dydrogesterone than progesterone. This observation highlights the need to reconsider the use of dydrogesterone and to investigate whether the potential increased risk is associated with specific doses, treatment duration, or specific pharmacological associations. Addressing these uncertainties is essential to guide safer clinical decisions and to better define the circumstances under which dydrogesterone may pose an increased risk to pregnancy outcomes.

## Authors’ roles

F.P. conceived the commentary and drafted the original version of the article. All authors critically revised the drafts of the manuscript and approved its final version.

## References

[hoae073-B1] Chakravarty BN , Shirazee HH, Dam P, Goswami SK, Chatterjee R, Ghosh S. Oral dydrogesterone versus intravaginal micronised progesterone as luteal phase support in assisted reproductive technology (ART) cycles: results of a randomised study. J Steroid Biochem Mol Biol 2005;97:416–420. 16213136 10.1016/j.jsbmb.2005.08.012

[hoae073-B2] Ganesh A , Chakravorty N, Mukherjee R, Goswami S, Chaudhury K, Chakravarty B. Comparison of oral dydrogestrone with progesterone gel and micronized progesterone for luteal support in 1,373 women undergoing in vitro fertilization: a randomized clinical study. Fertil Steril 2011;95:1961–1965. 21333984 10.1016/j.fertnstert.2011.01.148

[hoae073-B3] Griesinger G , Blockeel C, Kahler E, Pexman-Fieth C, Olofsson JI, Driessen S, Tournaye H. Dydrogesterone as an oral alternative to vaginal progesterone for IVF luteal phase support: a systematic review and individual participant data meta-analysis. PLoS One 2020;15:e0241044. 33147288 10.1371/journal.pone.0241044 PMC7641447

[hoae073-B4] Griesinger G , Blockeel C, Sukhikh GT, Patki A, Dhorepatil B, Yang DZ, Chen ZJ, Kahler E, Pexman-Fieth C, Tournaye H. Oral dydrogesterone versus intravaginal micronized progesterone gel for luteal phase support in IVF: a randomized clinical trial. Hum Reprod 2018;33:2212–2221. 30304457 10.1093/humrep/dey306 PMC6238366

[hoae073-B5] Griesinger G , Tournaye H, Macklon N, Petraglia F, Arck P, Blockeel C, van Amsterdam P, Pexman-Fieth C, Fauser BC. Dydrogesterone: pharmacological profile and mechanism of action as luteal phase support in assisted reproduction. Reprod Biomed Online 2019;38:249–259. 30595525 10.1016/j.rbmo.2018.11.017

[hoae073-B6] Haas DM , Hathaway TJ, Ramsey PS. Progestogen for preventing miscarriage in women with recurrent miscarriage of unclear etiology. Cochrane Database Syst Rev 2018;10:CD003511. 30298541 10.1002/14651858.CD003511.pub4 PMC6516817

[hoae073-B7] Heinonen OP , Slone D, Monson RR, Hook EB, Shapiro S. Cardiovascular birth defects and antenatal exposure to female sex hormones. N Engl J Med 1977;296:67–70. 830309 10.1056/NEJM197701132960202

[hoae073-B8] Henry A , SantullI P, Mathilde B, Maignien C, Chapron C, Treluyer JM, Guibourdenche J, Chouchana JL. Birth defects reporting and the use of dydrogesterone: a disproportionality analysis from the World Health Organization pharmacovigilance database (VigiBase). Hum Reprod Open 2024;2024:hoae072. 10.1093/hropen/hoae072 PMC11726828 39807112

[hoae073-B9] Katalinic A , Noftz MR, Garcia-Velasco JA, Shulman LP, van den Anker JN, Strauss JF. III, No additional risk of congenital anomalies after first-trimester dydrogesterone use: a systematic review and meta-analysis. Hum Reprod Open 2024;2024:hoae004. 10.1093/hropen/hoae004 PMC10859181 38344249

[hoae073-B10] Katalinic A , Shulman LP, Strauss JF, Garcia-Velasco JA, van den Anker JN. A critical appraisal of safety data on dydrogesterone for the support of early pregnancy: a scoping review and meta-analysis. Reprod Biomed Online 2022;45:365–373. 35644880 10.1016/j.rbmo.2022.03.032

[hoae073-B11] Manickavasagam M , Vakil A, Singh E, Roy H, Lal N, Patel RG, Mishra R, Gudibanda S, Shah S. Real-world evidence of dydrogesterone 20 mg and 30 mg SR usage in pregnancy. Cureus 2024;16:e72016. 39569237 10.7759/cureus.72016 PMC11577976

[hoae073-B12] Saharkhiz N , Zamaniyan M, Salehpour S, Zadehmodarres S, Hoseini S, Cheraghi L, Seif S, Baheiraei N. A comparative study of dydrogesterone and micronized progesterone for luteal phase support during in vitro fertilization (IVF) cycles. Gynecol Endocrinol 2016;32:213–217. 26486011 10.3109/09513590.2015.1110136

[hoae073-B13] Tomic V , Tomic J, Klaic DZ, Kasum M, Kuna K. Oral dydrogesterone versus vaginal progesterone gel in the luteal phase support: randomized controlled trial. Eur J Obstet Gynecol Reprod Biol 2015;186:49–53. 25622239 10.1016/j.ejogrb.2014.11.002

[hoae073-B14] Tournaye H , Sukhikh GT, Kahler E, Griesinger G. A phase III randomized controlled trial comparing the efficacy, safety and tolerability of oral dydrogesterone versus micronized vaginal progesterone for luteal support in in vitro fertilization. Hum Reprod 2017;32:1019–1027. 28333318 10.1093/humrep/dex023 PMC5400051

[hoae073-B15] Yu X , Nassar N, Mastroiacovo P, Canfield M, Groisman B, Bermejo-Sánchez E, Ritvanen A, Kiuru-Kuhlefelt S, Benavides A, Sipek A et al Hypospadias prevalence and trends in International Birth Defect Surveillance Systems, 1980-2010. Eur Urol 2019;76:482–490. 31300237 10.1016/j.eururo.2019.06.027 PMC7265200

[hoae073-B16] Zaqout M , Aslem E, Abuqamar M, Abughazza O, Panzer J, De Wolf D. The impact of oral intake of dydrogesterone on fetal heart development during early pregnancy. Pediatr Cardiol 2015;36:1483–1488. 25972284 10.1007/s00246-015-1190-9

